# Genetic Background Can Result in a Marked or Minimal Effect of Gene Knockout (GPR55 and CB_2_ Receptor) in Experimental Autoimmune Encephalomyelitis Models of Multiple Sclerosis

**DOI:** 10.1371/journal.pone.0076907

**Published:** 2013-10-09

**Authors:** Sofia Sisay, Gareth Pryce, Samuel J. Jackson, Carolyn Tanner, Ruth A. Ross, Gregory J. Michael, David L. Selwood, Gavin Giovannoni, David Baker

**Affiliations:** 1 Neuroimmunology, Blizard Institute, Barts and The London School of Medicine and Dentistry, Queen Mary University of London, London, United Kingdom; 2 School of Medical Science, Institute of Medical Sciences, University of Aberdeen, Foresterhill, Aberdeen, United Kingdom; 3 Pharmacology and Toxicology, University of Toronto, Toronto, Ontario, Canada; 4 Biological and Medical Chemistry, the Wolfson Institute for Biomedical Research, University College London, London, United Kingdom; San Raffaele Scientific Institute, Italy

## Abstract

Endocannabinoids and some phytocannabinoids bind to CB_1_ and CB_2_ cannabinoid receptors, transient receptor potential vanilloid one (TRPV1) receptor and the orphan G protein receptor fifty-five (GPR55). Studies using C57BL/10 and C57BL/6 (*Cnr2* ^tm1Zim^) CB_2_ cannabinoid receptor knockout mice have demonstrated an immune-augmenting effect in experimental autoimmune encephalomyelitis (EAE) models of multiple sclerosis. However, other EAE studies in Biozzi ABH mice often failed to show any treatment effect of either CB_2_ receptor agonism or antagonism on inhibition of T cell autoimmunity. The influence of genetic background on the induction of EAE in endocannabinoid system-related gene knockout mice was examined. It was found that C57BL/6.GPR55 knockout mice developed less severe disease, notably in female mice, following active induction with myelin oligodendrocyte glycoprotein 35-55 peptide. In contrast C57BL/6.CB_2_ (*Cnr2* ^Dgen^) receptor knockout mice developed augmented severity of disease consistent with the genetically and pharmacologically-distinct, *Cnr2* ^tm1Zim^ mice. However, when the knockout gene was bred into the ABH mouse background and EAE induced with spinal cord autoantigens the immune-enhancing effect of CB_2_ receptor deletion was lost. Likewise CB_1_ receptor and transient receptor potential vanilloid one knockout mice on the ABH background demonstrated no alteration in immune-susceptibility, in terms of disease incidence and severity of EAE, in contrast to that reported in some C57BL/6 mouse studies. Furthermore the immune-modulating influence of GPR55 was marginal on the ABH mouse background. Whilst sedative doses of tetrahydrocannabinol could induce immunosuppression, this was associated with a CB_1_ receptor rather than a CB_2_ receptor-mediated effect. These data support the fact that non-psychoactive doses of medicinal cannabis have a marginal influence on the immune response in MS. Importantly, it adds a note of caution for the translational value of some transgenic/gene knockout and other studies on low-EAE susceptibility backgrounds with inconsistent disease course and susceptibility.

## Introduction

Multiple sclerosis (MS) is an immune-mediated, demyelinating disease of the central nervous system. This results in the development of troublesome symptoms, some of which respond to treatment with cannabis [1]. The endocannabinoid system consists of CB_1_ and CB_2_ cannabinoid receptors and a number of endocannabinoid ligands and their synthetic and degradation molecules. However, endocannabinoids and phytocannabinoids may stimulate other receptors such as transient receptor potential vanilloid one (TRPV1) ion channel and the orphan G protein coupled receptor 55 (GPR55) [2-5]. There is increasing evidence that Δ^9^-tetrahydrocannabinol (THC. A CB_1_ and CB_2_ receptor agonist [2]) within cannabis and the CB_1_ receptor can regulate aberrant synaptic neurotransmission and control symptoms such as spasticity, which are associated with nerve damage in MS [1,2,6]. Pharmacological control of spasticity translated from experimental autoimmune encephalomyelitis (EAE) model in rodents to the treatment of MS in humans [1,7,8]. However, the distribution of CB_1_ receptors on nerves and CB_2_ receptors on immune cells [2] suggest that cannabinoids may have additional influences on MS. As such there is increasing evidence to indicate that cannabinoids may control neurodegenerative mechanisms [9,10]. There is also much interest on whether cannabinoids may influence (auto)immune aspects of MS that may drive relapsing disease. 

There are no cannabinoid receptor ligands that have total receptor specificity; rather all have varying degrees of receptor selectivity [11]. *In vitro* assays allow dose-titration and off-target effects to be minimised, but *in vivo* this may be more complex where high doses may be administered to get adequate receptor coverage over time. However, depending on the bioavailability and route of administration there may be high peaks of compound concentration and drug metabolism has the potential of creating new active molecules. Both of these factors increase the chance of off-target effects. Whilst target validation is often achieved by use of pharmacological antagonists, these too have off-target effects [11]. Thus, specific gene deletion or gene silencing provides an extra level of precision in determining target validity [6]. The influence of cannabinoid receptor deletion in the initial acute phase of disease models of MS has been reported previously for CB_1_ receptor [9,10,12], CB_2_ receptor [12,13] and TRPV1 gene knockout mice [14]. The influence of GPR55 on EAE is however unknown. GPR55 is expressed at low levels in a variety of tissues that include blood vessels and nervous tissue and immune tissues. However, the function of GPR55 is poorly defined [3-5]. This study examined the influence of GPR55 gene knockout on susceptibility to EAE.

Initial studies in EAE using central nervous system myelin and myelin basic protein indicated that susceptibility was polygenic with an important influence of major histocompatibility complex (MHC) haplotype. It was found that C57BL/6 and 129 mice (H-2^b^) are relatively EAE resistant compared to highly susceptible strains such as SJL (H-2^s^) and Biozzi ABH (H-2^dq1^) mice [15,16]. However, the demonstration that myelin oligodendrocyte glycoprotein (MOG) could induce disease in H-2^b^ mice [17] means that the majority of studies using transgenic and gene knockout tissue are now performed in MOG_35-55_ peptide-induced EAE in C57BL/6 mice. Previously we have reported that CB_2_ knockout C57BL/10.Cnr2^tm1Zim^ mice develop augmented EAE, yet pharmacological agonism and antagonism of CB_2_ receptors consistently failed to influence the development of EAE, when examined in ABH mice [12,18]. Disease in C57BL/6 can be highly variable in terms of timing of onset, and the disease severity induced [19,20]. Therefore, we hypothesised that the immune-modulating influence of CB_2_ deficiency may be lost when studies are performed in strains that are fully susceptible to EAE induction. The influence of cannabinoid gene deletion on an EAE susceptible background was examined and demonstrated that they have a limited immune phenotype, which affects susceptibility to disease induction. 

## Materials and Methods

### Ethics Statement

All animal studies were approved by the Queen Mary University Ethical Review panel and the United Kingdom Government Home Office Inspectorate. These studies where performed under Licence from the UK Home Office and conformed to the United Kingdom Animals (Scientific Procedures) Act 1986 for the use of animals in research.

### Animals

Mice were from in-house bred stock that was maintained in a 12h light/dark cycle with controlled humidity and temperature and animals were fed RM-1E diet and water *ad libitum*. These were housed as described previously, to conform with the ARRIVE guidelines [21]. Biozzi ABH and congenic ABH.*Cnr1* ^*tm1Par*^ mice CB_1_ receptor knockout mice [9] were from stock bred at Queen Mary University of London. 129 mice were purchased from Charles Rivers, Margate, UK. Male B6.129P2-*Cnr2^tm1Dgen^/J* homozygous CB_2_ receptor knockout mice [22] were purchased from Jackson Laboratories (Bar Harbor, Maine USA). These mice had been backcrossed onto the C57BL/6/J background for more than 5 generations at the time of arrival. These were backcrossed with C57BL/6/J (Charles Rivers Margate, Kent) and then intercrossed to produce C57BL/6.*Cnr2* ^*tm1Dgen*^ CB_2_ receptor knockout mice. In addition, mice were backcrossed with ABH mice for more than 11 generations, screening for CB_2_ receptor expression at each generation, prior to intercross to produce congenic ABH.*Cnr2* ^*tm1Dgen*^ mice. C57BL/6.*Trpv1* ^*tm1Jbd*^, TRPV1 knockout mice [23] were obtained from Dr. John B Davis, Glaxo Smith Kline, Stevenage, UK. These were backcrossed with ABH mice for 6 generations and screened for the expression of the neomycin resistance gene [9] within the targeting expression cassette at each generation, prior to intercross to produce ABH.*Trpv1* ^*tm1Jbd*^ mice. Functional knockout of the gene was demonstrated following the lack visible sedation and lack of hypothermia (>1°C body temperature loss within 20min following injection i.v. of 0.5mg/kg arvanil in measured with a thermocouple as described previously [21]). Loss of receptor expression was confirmed by TRPV1-specific immunocytochemistry [24]. Founder B6.129-Gpr55^tm1Tigm^ GPR55 knockout mice were purchased from the Texas Institute of Genomic Medicine (College Station, Texas, USA). This mouse lacks the entire coding sequence as described [25]. These were caesarean re-derived and backcrossed twice with C57BL/6/J mice. Then heterozygous animals were crossed with either a heterozygous or homozygous mouse to produce C57BL/6.Gpr55-/-. In addition mice were backcrossed with ABH mice for over 11 generations to produce ABH.*Gpr55* ^tm1Tigm^ mice and following intercross ABH.Gpr55^-/-^ were generated. Wildtype C57BL/6/J and C57BL/6.*Cnr1* ^*tm1Zim*^ CB_1_ receptor knockout mice [26], C57BL/6.*Cnr2* ^*tm1Zim*^CB_2_ receptor knockout mice [27] were obtained from Dr. George Kunos, National Institutes of Health, Bethesda, Maryland, USA, C57BL/6.*Cnr2* ^*tm1Dgen*^ CB_2_ receptor knockout mice and C57BL/6.Gpr55^tm1Tigm^ GPR55 knockout mice derived from the stock above were bred in individual ventilated cages at the University of Aberdeen.

### Chemicals

Arvanil (N-Vanillylarachidonamide) was purchased from Cayman Chemical (AnnHarbor, Michigan, USA). R(+) WIN55 212-2 (WIN55.(R)-(+)-[2,3-Dihydro-5-methyl-3-(4-morpholinylmethyl)pyrrolo[1,2,3-*de*]-1,4-benzoxazin-6-yl]-1-naphthalenylmethanone mesylate) was purchased from Tocris, Bristol, UK. Δ^9^Tetrahydrocannabinol (THC) was purchased from THC pharm, Frankfurt, Germany. A selective GPPR55 ligand (R)3-(5-dimethylcarbamoyl-pent-1-enyl)-N-(2-hydroxy-1-methyl-ethyl) benzamide was synthesised as described previously [28]. 2007). WIN55 was dissolved in dimethyl sulphoxide:cremaphor:phosphate buffered saline (PBS) 1:1:18. These were purchased from Sigma (Poole, Dorset, UK). Arvanil and THC were dissolved in ethanol:cremophor:PBS (1:1:18). These were administered via the intravenous route, for screening purposes, or daily via the intraperitoneal routes in 0.1ml.

### Genotyping

Genotyping was performed as described previously [9] Briefly ear biopsies were removed from weaned mice and DNA samples were prepared following digestion overnight at 60°C in 500 μl 0.2g/ml Proteinase K (Invitrogen, Paisley, UK) in Nucleon TM Reagent B lysis buffer pH8 (400mM Tris/HCl, 60mM EDTA, NaCl 150mM, 1 % sodium dodecyl sulphate). 150μl 5M sodium perchlorate was added followed by a further 30 minute incubation at 60°C. Equal volumes of chloroform were added, the sample vortexed and centrifuged for 4 minutes at 14000rpm in an Eppendorf microfuge. The aqueous phase was added to 2 volumes of cold ethanol to precipitate the DNA, which was then dissolved in water. DNA was amplified using polymerase chain reaction using Qiagen PCR core kit reagents (Qiagen, Crawley, UK) as described previously [9]. Samples were amplified using 35 cycles 94°C 60s, 55 or 60°C 60s, 72°C 60s) with *Cnr2* primers (Annealing temperature 60°C, Forward 5’GGGGATCGATCCGTCCTGTAAGTCT3’, Reverse1 5’GGAGTTCAACCCCATGAAGGAGTAC3’, Reverse2 5’GACTAGAGCTTTGTAAGGTAGGC3’. Size of products: wildtype 350 base pairs, transgene 500 base pairs) and *Gpr55* primers (Annealing temperature 55°C, Forward 5’TCTGGATTCATCGACTGTG3’, Reverse1 5’TCCACAATCAAGCTG3’, Reverse 2. 5’GTCACCCATCCAGGTGAT3’. Size of product: wildtype 207 base pairs and transgene 299 base pairs). Products were identified by gel electrophoresis using 2% agarose in Tris borate EDTA buffer (Sigma) gels 

### CB_2_ receptor Binding Assays

The binding affinity of compounds was performed by contract research organisations on stably human *CNR2* transfected cell lines using cyclic AMP assays (Multispan Inc. Hayward, California, USA) or GTPγS binding assay (MDS pharma. Taipei, Taiwan).

### Vas Deferens Assay

Vasa deferentia were obtained from mice weighing 30 to 50 g. Each tissue was mounted in a 4 ml organ bath at an initial tension of 0.5g. The baths contained Mg^2+^-free Krebs solution which was kept at 35°C and bubbled with 95% O_2_ and 5% CO_2_. The composition of the Krebs solution was (mM): NaCl 118.2, KCl 4.75, KH_2_PO_4_ 1.19, NaHCO_3_ 25.0, glucose 11.0 and CaCl_2_ .6H_2_O 2.54. Isometric contractions were evoked by stimulation with 0.5s trains of three pulses of 110% maximal voltage (train frequency 0.1Hz; pulse duration 0.5ms) through a platinum electrode attached to the upper end and a stainless steel electrode attached to the lower end of each bath. Stimuli were generated by a Grass S48 stimulator, then amplified (Med-Lab channel attenuator) and divided to yield separate outputs to four organ baths (Med-Lab StimuSplitter). Contractions were monitored by computer using a data recording and analysis system (MacLab) that was linked via preamplifiers (Macbridge) to UF1 transducers. After placement in an organ bath, each tissue was subjected to a stimulation-free period of 15 min and then stimulated for 10 min. Tissues were then subjected to alternate periods of stimulation (5 min) and rest (10 min) until consistent twitch amplitudes were obtained. This equilibration procedure was followed by a stimulation-free period of 30 min. Tissues were then stimulated for 10 min after which the stimulator was switched off and (R)3-(5-dimethylcarbamoyl-pent-1-enyl)-N-(2-hydroxy-1-methyl-ethyl)benzamide or its vehicle added. Additions of the compounds were made cumulatively at 15 min intervals without washout, the tissues being stimulated for the final two minutes of exposure to each concentration of this agonist. Compounds were dissolved in DMSO at 10mM and diluted in saline. By themselves, these vehicles did not inhibit the twitch response. Drug additions were made in a volume of 10 µl. R(+) WIN55,212 served as a positive control. The degree of inhibition of evoked contractions induced by agonist was calculated in percentage terms by comparing the amplitude of the twitch response after each addition of agonist with its amplitude immediately before the first addition of this agonist [29].

### Induction of Experimental Autoimmune Encephalomyelitis

ABH mice and ABH congenic mice were injected with 1mg of freeze dried spinal cord homogenate in Freunds adjuvant in the flank on day 0 and 7 as described previously [21]. C57BL/6 wildtype and C57BL/6-transgenic mice were injected with 200µg mouse MOG_35-55_ peptide in Freunds adjuvant supplemented with 400µg/mouse *Mycobacterium tuberculosis* H37Ra on day 0 and day 7. These were also injected i.p. with 200ng *Bordetella pertussis* toxin (Sigma) in phosphate buffered saline at the time of administration of the Freunds adjuvant and this was repeated after 24h as described previously [21,30]. Randomisation for allocation to groups, sample size calculations, blinding and other aspects of experimental design and reporting consistent with the ARRIVE guidelines have been described previously [21]. Disease was monitored and scored 0=normal, 1=limp tail, 2=impaired righting reflex, 3= paresis of the hind limbs, 4=hind limb paralysis, 5= moribund (endpoint)/death with 0.5 less than the indicated grade for milder signs as described previously [21]. The data is presented as the mean daily clinical score ±standard error of the mean (SEM) or the mean maximal clinical score of the group (Group Score) ±SEM; the mean maximal clinical score of the animals that developed clinical disease (EAE Score) ±SEM and the mean day of onset ± standard deviation (SD). Differences between groups, including disease incidence, were assessed using non-parametric, Mann Whitney U statistics using Sigmastat/Sigmaplot Software (Systat Chicago, Illinois, USA).

### Immunophenotyping

Mice were killed by CO_2_ overdose or by cervical dislocation and lymphocytes from blood and spleen were collected under sterile conditions. Large spleen fragments were initially removed by passing the cell suspension though a nylon mesh on a 50 ml falcon tube and the cells were recovered by centrifugation for 5 min at 478g. Erythrocytes from the spleen cell suspension and from the blood were then lysed with a hypotonic ammonium chloride red blood cell lysis buffer (eBioscience Ltd, Hatfield, UK) for 5 min at room temperature. 100μl of 2x10^6^ cells/ml in staining buffer (1xPBS, 2% foetal calf serum) were incubated with various antibodies to surface antigens (CD3, CD4, CD8, CD19, CD11c, CD25, CD45 and F4/80) and intracellular cytokines (IL-2, IL-4, IL-10, IL-17A and IFN-γ) (BD bioscience, Oxford, UK). Antibodies were diluted 1:100 and incubation conducted for 30 min in the dark at 4°C or on ice. A transcription factor FOX3P was also used to identify regulatory T cells. After incubation, 3ml of staining buffer was added to each tube. Tubes were then centrifuged at 478g 5 min at 4°C. After centrifugation pellets were resuspended in 300μl of staining buffer. Cells were analysed using flow cytometry.

The production of cytokines were supported using quantitative polymerase chain reaction to detect IL-2 Forward 5’GCATGTTCTGGATTTGACTC3’ and reverse 5’CAGTTGCTGACTCATCATCG3; IL-4 Forward 5’CAAACGTCCTCACAGCAACG3’ and reverse 5’CTTGGACTCATTCATGGTGC3’; IL-10 Forward 5’GGTTGCCAAGCCTTATCGGA3’ and reverse 5’ACCTGCTCCACTGCCTTGCT3’; IL-17A Forward5’AGCGTGTCCAAACACTGAGG3’ and reverse 5’CTATCAGGGTCTTCATTGCG3’; Interferon gamma Forward5’CCATCAGCAACAACATAAGC3’ and reverse 5’AGCTCATTGAATGCTTGGCG3’; and beta actin Forward 5’AATCGTGCGTGACATCAAAG3’ and reverse 5’ATGCCACAGGATTCCATACC3’. Quantitative PCR was performed in duplicates in 96-well reaction plates with the Applied Biosystems 7500 Real-Time PCR system (Applied Biosystems, Warrington, Cheshire, UK) and the cycling conditions for the qPCR were as follows: 95°C (15 min), 40 cycles of 94°C for (45 s), 58°C for (45 s), 72°C (40s).

Lymphocytes were collected on day 9 and re-stimulated *in vitro* with MOG peptide at concentrations 1μg/ml or 10μg/ml for 72h. Lymphocytes from naïve GPR55 knockout and wildtype mice were also collected and stimulated with 5μg/ml concanavalin A for 48h. A total of 300,000 cells were then resuspended in a final volume of 100μl of RPMI, 10% FCS and plated in 96 well-plates. A total of 0.5 units of ^3^H thymidine (PerkinElmer LAS, Beaconsfield, Bucks, UK) was added to each well and cells were incubated during for 24h at 37°C in 5%CO_2_. Cells were then harvested (Mach III M cell harvester 96, Tomtec, Warwick UK) and analysed on a counter (Wallac 1450, Microbeta plus Liquid Scintillation Counter, Cambridgeshire, UK). In some instances proliferation was assessed using 5μM carboxyfluorescein diacetate, succinimidyl ester (Invitrogen, Paisley, UK), which was incubated with cells at 37°C for 10 minutes. One volume of ice-cold foetal calf serum (FCS) was then added to quench the staining and cells were then washed twice in staining buffer (1x PBS 2% FCS) and then incubated for 4 days. Samples were analysed by flow cytometry.

## Results

### GPR55 deficient C57BL/6 mice have an immunophenotype in female mice

**Figure 1:**
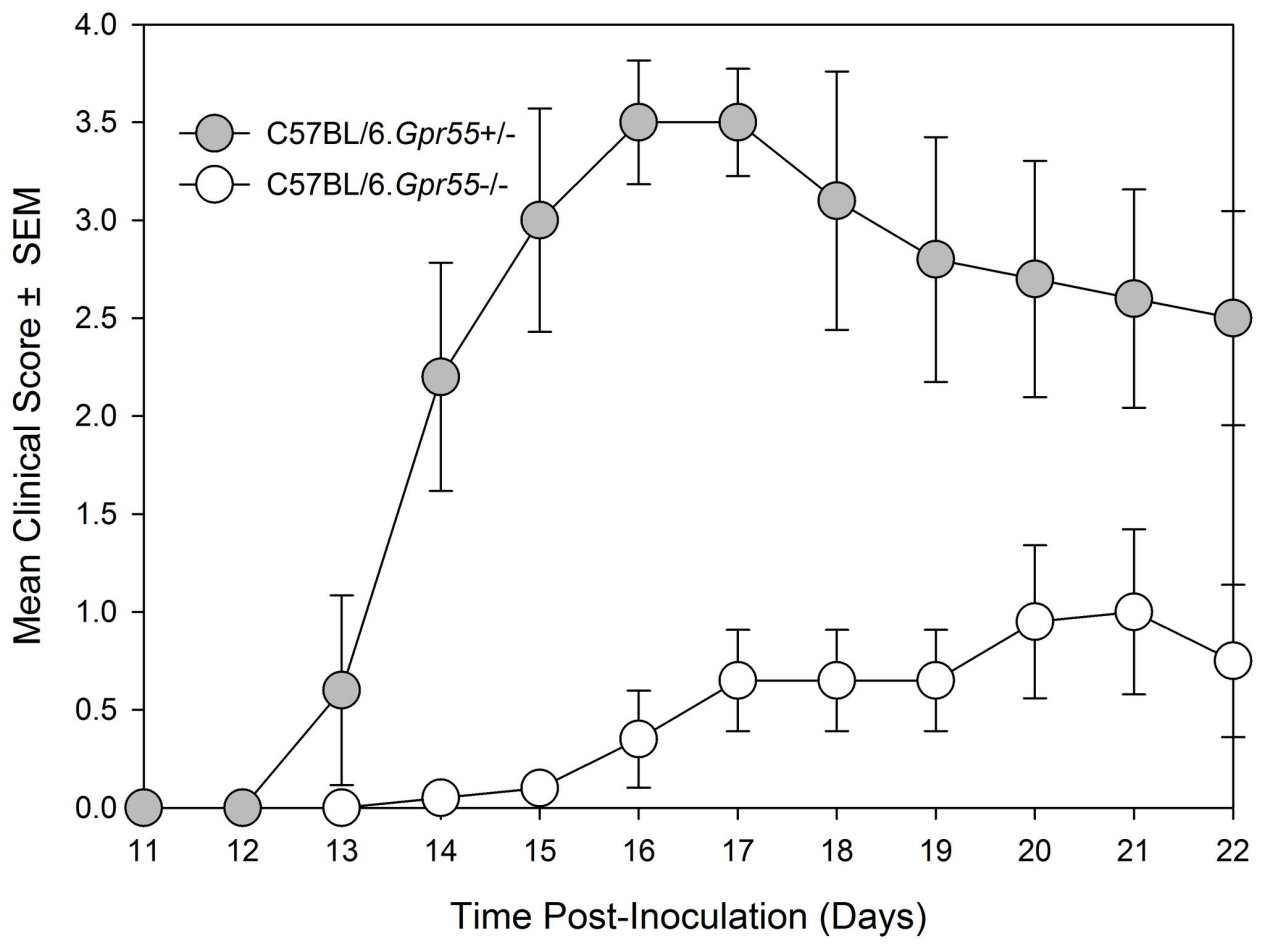
GPR55-deficiency can inhibit the development of EAE in C57BL/6 mice. Female C57BL/6 GPR55 knockout (n=10 white circles) and heterozygous wildtype littermates (n=5. Filled grey circles) were injected with 200μg MOG_35-55_ peptide in Freunds adjuvant on day 0 and 7. Neurological signs were scored daily 0-5 scale. The results represent the mean ± SEM daily scores (Table 1A).

Founder mice with the Gpr55^tm1Tigm^ transgene that deletes the entire coding region of Gpr55 were obtained and backcrossed onto the C57BL/6 background. Following immunization of mice with MOG_35-55_ peptide in Freunds adjuvant, it was found that GPR55-deficient mice developed significantly (P<0.05) lower severity disease compared to their littermates (Figure 1. Table 1A). As males were being used for analysis in vas deferens assays female animals were used. Therefore, it was of interest when further analysis of additional GPR55-deficient mice was undertaken the data indicated that female mice were notably more resistant to EAE induction than male mice. Only 2/8 female Gpr55-/- mice developing EAE with a group score 1.0 ± 0.7 compared with 11/12 wildtype littermates developing EAE with a score of 2.9 ± 0.4 (P<0.05). (Table 1B) Disease in male GPR55 knockout mice was not significantly different from wildtype littermates with a group score 2.5 ± 0.8 compared to 3.0 ± 0.5 in littermates. These mice did not relapse or develop spasticity. These data suggest that there is an immune phenotype in these mice that inhibits the generation of T cell autoimmunity. There was however, no apparent differences in the number of CD4+, CD8+, CD19+, CD11c+, F4/80+ and CD4+, Fox3P+, CD25+ regulatory cells in the thymus, spleen and blood (n=5/group) of male and female C57BL/6.Gpr55-/- and wildtype mice assessed using flow cytometry. Furthermore there were no differences in their mitogenic T cell responses to 5µg concanavalin A in male or female mice (Figure S1A). Mice failed to give specific-proliferative responses to MOG_35-55_ *in vitro* even after immunization (Figure S1B), because there was a high endogenous proliferation in splenocytes from animals injected with MOG_35-55_ peptide in Freunds adjuvant and pertussis toxin (Figure S2). No specific proliferation was detected irrespective of GPR55 genotype in male or female (Figure S2). There were no differences in gamma interferon, IL-4, IL-10 or IL-17 levels (data not shown). 

**Table 1:** CB_1_, CB_2,_ TRVP1 and GPR55 gene deletions exhibit minimal impact on the develop of an autoimmune response on the ABH mouse background compared to C57BL/6 background.

Code	Strain	No. EAE	Group Score ± SEM	EAE Score ± SEM	Day of Onset ± SD
	**Initial Acute Disease**				
A	♀C57BL/6.*Gpr55*-/+	5/5	3.6 ± 0.3	3.6 ± 0.3	13.8 ± 0.8
	♀C57BL/6.*Gpr55*-/-	5/10	1.1 ± 0.4**	2.1 ± 0.5*	15.8 ± 1.7*
	♀C57BL/6.*Cnr2*+/+	4/9	0.9 ± 0.5	2.0 ± 0.7	15.3 ± 2.2
	♀C57BL/6.*Cnr2-/-*	9/10	3.3 ± 0.4**	3.7 ± 0.2	16.8 ± 0.5*
B	♂C57BL/6.*Gpr55*+/+	11/12	3.0 ± 0.5	3.9 ± 0.1	15.3 ± 1.1
	♂C57BL/6.*Gpr55*+/+	4/6	2.5 ± 0.8	3.8 ± 0.8	18.0 ± 1.4*
	♀C57BL/6.*Gpr55*-/+	11/12	2.9 ± 0.4	3.1 ± 0.4	16.2 ± 1.3
	♀C57BL/6.*Gpr55*-/-	2/8**	1.0 ± 0.7*	4.0 ± 0.0	15.0 ± 1.9
C	♀C57BL/6.*Cnr2*+/+	1/10	0.1 ± 0.1	1.0 ± n/a	17.0 ± n/a
	♀C57BL/6.*Cnr2-/-*	6/11	1.3 ± 0.4	2.4 ± 0.3	16.8 ± 1.7
	♀129 wildtype	9/10	1.0 ± 0.1	1.1 ± 0.1	16.6 ± 0.7
	♀ABH wildtype	14/18	3.0 ± 0.4	3.8 ± 0.1	16.6 ± 2.2
D	♂♀ABH.*Cnr2*+/+	13/13	4.5 ± 0.1	4.5 ± 0.1	15.7 ± 0.8
	♂♀ABH.*Cnr2*-/-	12/12	4.2 ± 0.2	4.2 ± 0.2	15.8 ± 1.9
E	♂♀ABH.*Cnr1*+/+	6/6	4.0 ± 0.0	4.0 ± 0.0	16.3 ± 1.8
	♂♀ABH.*Cnr1*-/-	15/15	4.1 ± 0.1	4.1 ± 0.1	16.3 ± 1.8
F	♂♀ABH.*Trpv1*+/+	9/9	3.6 ± 0.1	3.6 ± 0.1	15.4 ± 1.1
	♂♀ABH.*Trpv1*-/-	11/11	3.8 ± 0.1	3.8 ± 0.1	16.0 ± 1.0
G	♂ABH.*Gpr55*+/+	16/16	3.6 ± 0.1	3.6 ± 0.1	16.6 ± 2.2
	♂ABH.*Gpr55-/-*	12/13	3.2 ± 0.3	3.2 ± 0.3	17.1 ± 2.0
	♀ABH.*Gpr55*+/+	12/12	3.8 ± 0.1	3.8 ± 0.1	15.2 ± 1.2
	♀ABH.*Gpr55-/-*	21/21	3.1 ± 0.2*	3.1 ± 0.2*	16.6 ± 2.4
	**Induced Relapse**				
H	♂ABH.*Gpr55*+/+	16/16	3.9 ± 0.1	3.9 ± 0.1	34.9 ± 1.4
	♂ABH.*Gpr55-/-*	13/13	3.7 ± 0.2	3.7 ± 0.2	34.7 ± 1.4
	♀ABH.*Gpr55*+/+	12/12	3.9 ± 0.1	3.9 ± 0.1	35.4 ± 1.3
	♀ABH.*Gpr55-/-*	21/21	3.8 ± 0.1	3.8 ± 0.1	34.8 ± 1.0

Animals were injected with either mouse spinal homogenate in Freunds adjuvant in ABH mice or MOG_35-55_ peptide in Freunds adjuvant on day 0 and 7and PTX in C57BL/6 background mice. The results show the incidence of EAE, the mean maximal neurological score for the group ± SEM, score of animals that develop clinical EAE ± SEM and the first day of onset of neurological signs up to day 22 post-inoculation.

* P<0.05, **P<0.01 compared to littermate controls.

### CB_2_ receptor-deficient mice develop an augmented immune response on the C57BL/6 but not ABH mouse background.

As C57BL/6.GPR55 knockout mice, as shown here, and C57BL background CB_2_ (*Cnr2* ^tm1Zim^) knockout mice reported previously [12,13] show differences in EAE susceptibility, the following data prompted us also to reinvestigate the influence of CB_2_ receptor deletion on EAE. One of first compounds that was reported to stimulate GPR55 was (R) 3-(5-dimethylcarbamoyl-pent-1-enyl)-N-(2-hydroxy-1-methyl-ethyl) benzamide [28]. This could potently inhibit electrically induced, autonomic nerve-induced activity in the vas deferens (Figure S3). In contrast to effects in wildtype mice its efficacy was markedly absent when tested in the vas deferens assay from GPR55 knockout mice. This supported an effect of the compound at GPR55. The potent inhibition of contraction in wildtype mice (EC_50_ = 10.4nM) was essentially unaltered in CB_1_ receptor (*Cnr1* ^*tm1Zim*^) knockout mice (EC_50_ = 12.7nM). Likewise there was essentially no inhibitory effect in C57BL/6.Cnr2^tm1Dgen^, CB_2_ receptor knockout mice (EC_50_ = 17.6 nM). In contrast, the activity of (R) 3-(5-dimethylcarbamoyl-pent-1-enyl)-N-(2-hydroxy-1-methyl-ethyl) benzamide was markedly attenuated in C57BL/6.Cnr2^tm1Zim^ mice with a EC_50_ = 249.7nM (Figure S3). This was surprising as 3-(5-dimethylcarbamoyl-pent-1-enyl)-N-(2-hydroxy-1-methyl-ethyl)benzamide does not appear to bind to CB_2_ receptors, tested to 10µM, in stably transfected HEK293T.*CNR2* and CHO-K1,*CNR2*cells using cAMP assays (CP55,940 EC_50_=1nM) or GTPγS binding assays (CP55,940 EC_50_=2.37nM, WIN-55 EC_50_=2.37nM). This indicates that the two different CB_2_ receptor knockout mouse strains do not respond identically to pharmacological treatments and one may possibly have a defect in GPR55 function in addition to a functional silencing of CB_2_ receptor. Therefore, further EAE studies in C57BL/6.*Cnr2* ^tm1Zim^ mice were terminated.

**Figure 2:**
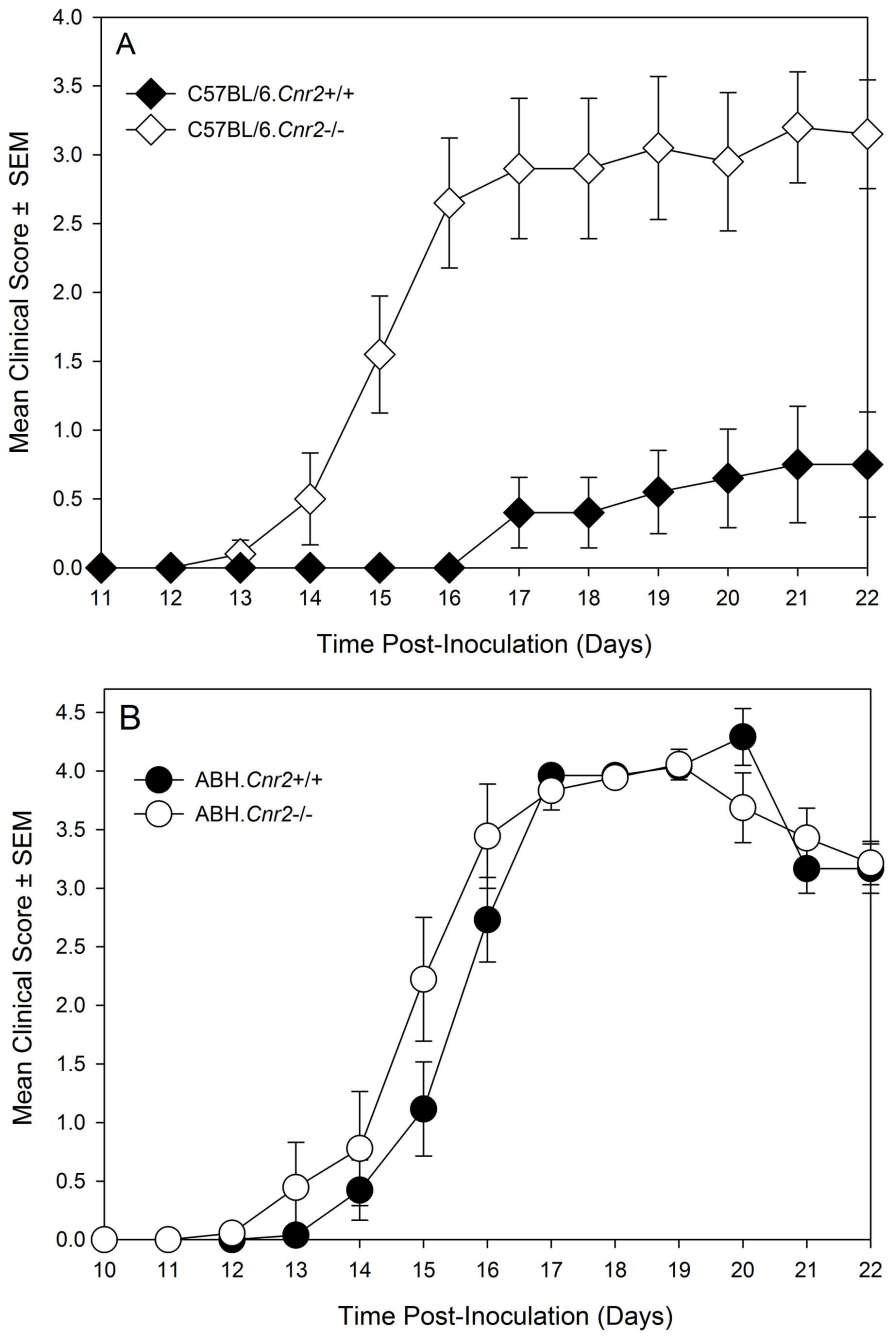
Genetic Background may influence the development of EAE in CB_2_ receptor knockout mice. (A) Female C57BL/6/J wildtype (black symbols. n= 9) and C57BL/6.*Cnr2* ^*tm1Dgen*^ CB_2_ receptor knockout mice (white symbols n=10) were injected with MOG_35-55_ peptide in Freunds adjuvant and PTX as co-adjuvant (Table 1B). (B) Male and Female wildtype ABH (black symbols n= 13) or ABH.*Cnr2* ^*tm1Dgen*^ (Grey symbols. n=12) CB_2_ receptor knockout mice were injected with spinal cord homogenate in Freunds adjuvant on day 0 & 7 (Table 1D). These were injected with vehicle or 25mg/kg i.p. THC daily from day 10 onwards (Table 2B). Neurological signs were scored daily 0-5 scale. The results represent the mean ± SEM daily scores.

**Figure 3:**
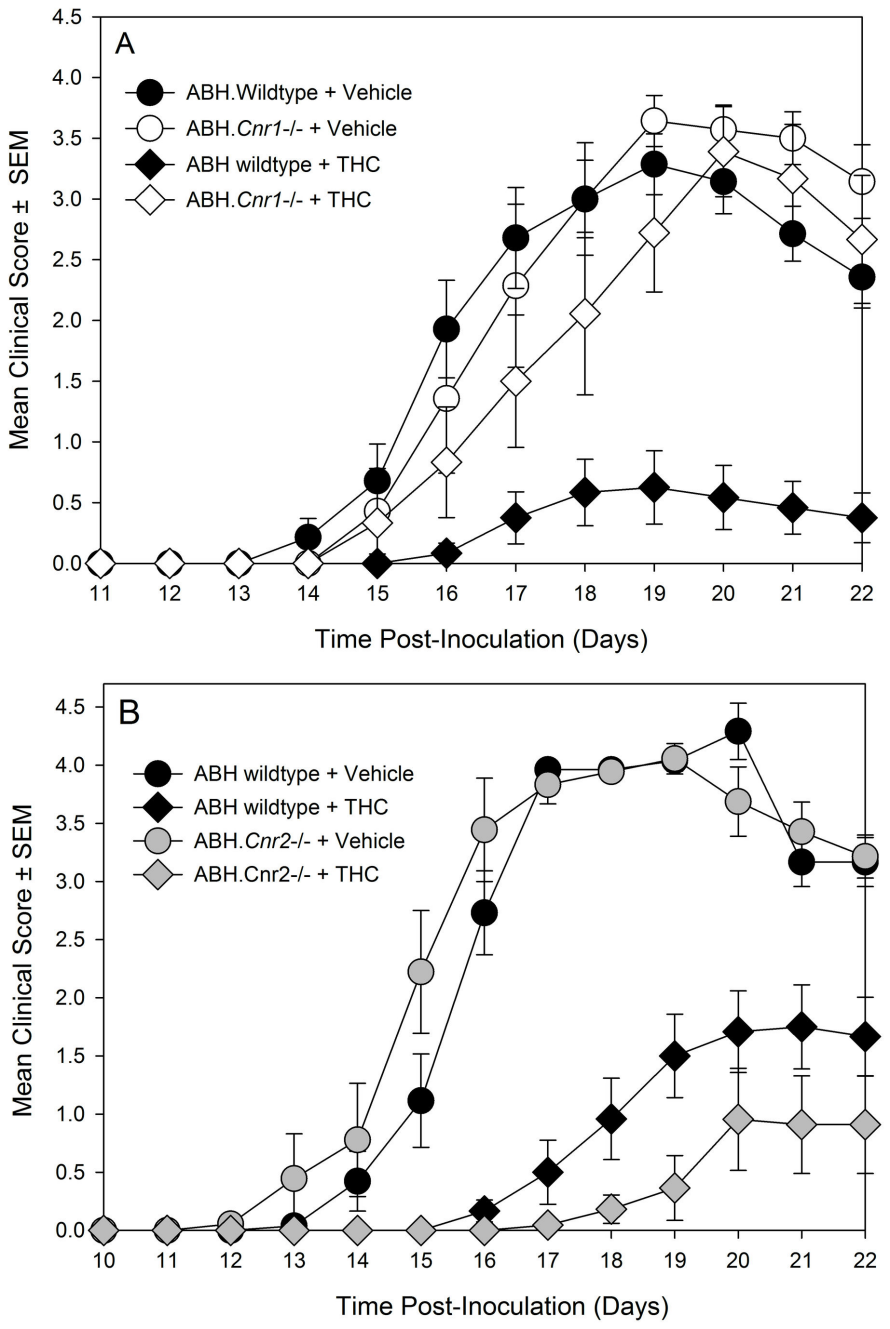
High doses of tetrahydrocannabinol inhibit autoimmunity in EAE via a CB_1_ receptor-dependent rather than a CB_2_ receptor-dependent mechanism. Wildtype (solid symbol) and CB_1_ receptor (Cnr1^tm1Par^. White symbol. Table 2A) and CB_2_ (Cnr2^tm1Dgen^. Grey symbol. Table 2B) receptor ABH congenic knockout mice were injected with mouse spinal homogenate in Freunds adjuvant on day 0 and 7. Animals were injected daily i.p. with 20-25mg/kg THC (Diamond symbol) in ethanol:cremophor:phosphate buffered saline (1:1:18) or vehicle (round symbol) in 0.1ml. The results show the incidence of EAE, the mean maximal neurological score for the group ± SEM, score of animals that develop clinical EAE ± SEM and the day of onset of neurological signs. *P<0.05, **P<0.01, ***P<0.001 compared to littermate controls.

New experiments were initiated in C57BL/6.Cnr2^tm1Dgen^ CB_2_ receptor knockout mice to determine whether they would respond similarly to C57BL/6.*Cnr2* ^tm1Zim^ CB_2_ receptor knockout mice. Indeed, it was found that C57BL/6.Cnr2^tm1Dgen^ CB_2_ receptor knockout developed more severe neurological signs (P<0.05) compared to wildtype littermates (Figure 2A and Table 1B, Table 1C). When EAE susceptibility was re-investigated following the production of fully congenic CB_2_ receptor deficient ABH mice, it was found that they exhibited a disease course that was comparable to wildtype ABH mice (Figure 2B and Table 1D). This lack of apparent influence of immune activity in ABH.CB_2_ knockout mice was seen also in CB_1_ (ABH.*Cnr1* ^tm1Par^) receptor (Table 1E, Figure 3A) and TRPV1 (ABH.*Trpv1* ^tm1Dav^) knockout mice (Table 1F). Previously, it has been reported that only doses above 2.5mg/kg THC i.p./day that induce cannabimimetic effects can induce immunosuppression in ABH mouse EAE [18]. It was evident that high dose (daily 20mg/kg i.p. Figure 3A or 25mg/kg i.p. Figure 3B) of THC, which caused visible sedation, could be immunosuppressive and significantly (P<0.001) inhibited the development and severity of EAE (Figure 3, Table 2). However, the immunosuppressive effect did not appear to be CB_2_ receptor mediated, but was largely a product of CB_1_ receptor activity. The immunosuppressive activity was markedly attenuated in CB_1_ receptor-deficient mice (Figure 3A) and was essentially unaltered in CB_2_ receptor knockout mice following daily administration of 25mg/kg i.p. (Figure 3B. Table 2). This further suggests that CB_2_ receptor agonism may have a weak potential to modulate robust T cell-driven inflammation. 

**Table 2:** High Dose of tetrahydrocannabinol inhibits autoimmunity in EAE via a CB_1_ receptor-dependent rather than a CB_2_ receptor-dependent mechanism.

Code	Strain	Treatment	No. EAE	Group Score ± SEM	EAE Score ± SEM	Day of Onset ± SD
A	ABH wildtype	Vehicle	14/14	3.7 ± 0.1	3.7 ± 0.1	16.2 ± 1.4
	ABH wildtype	THC	5/12*	0.5 ± 0.3***	1.5 ± 0.5**	17.0 ± 0.7
	ABH.*Cnr1*-/-	Vehicle	7/7	3.9 ± 0.1	3.9 ± 0.1	16.3 ± 1.1
	ABH.*Cnr1*-/-	THC	9/9	3.6 ± 0.4	3.6 ± 0.4	17.6 ± 1.7
B	ABH wildtype	Vehicle	13/13	4.5 ± 0.1	4.5 ± 0.1	15.2 ± 1.3
	ABH wildtype	THC	9/12	1.8 ± 0.4***	2.4 ± 0.3***	2.4 ± 0.3***
	ABH.*Cnr2*-/-	Vehicle	12/12	4.2 ± 0.2	4.2 ± 0.2	15.5 ± 2.1
	ABH.*Cnr2*-/-	THC	4/13**	0.8 ± 0.4***	2.5 ± 0.5**	18.8 ± 1.5*

Animals were injected with mouse spinal homogenate in Freunds adjuvant on day 0 and 7. Mice were injected daily i.p. with 20-25mg/kg THC i.p. in ethanol:cremophor:phosphate buffered saline (1:1:18) in 0.1ml. Animals were monitored from day 11-day 22 post-inoculation. The results show the incidence of EAE, the mean maximal neurological score for the group ± SEM, score of animals that develop clinical EAE ± SEM and the day of onset of neurological signs. *P<0.05, **P<0.01, ***P<0.001 compared to littermate controls.

### GPR55 deficient ABH mice have a very modest immunophenotype in female mice.

**Figure 4:**
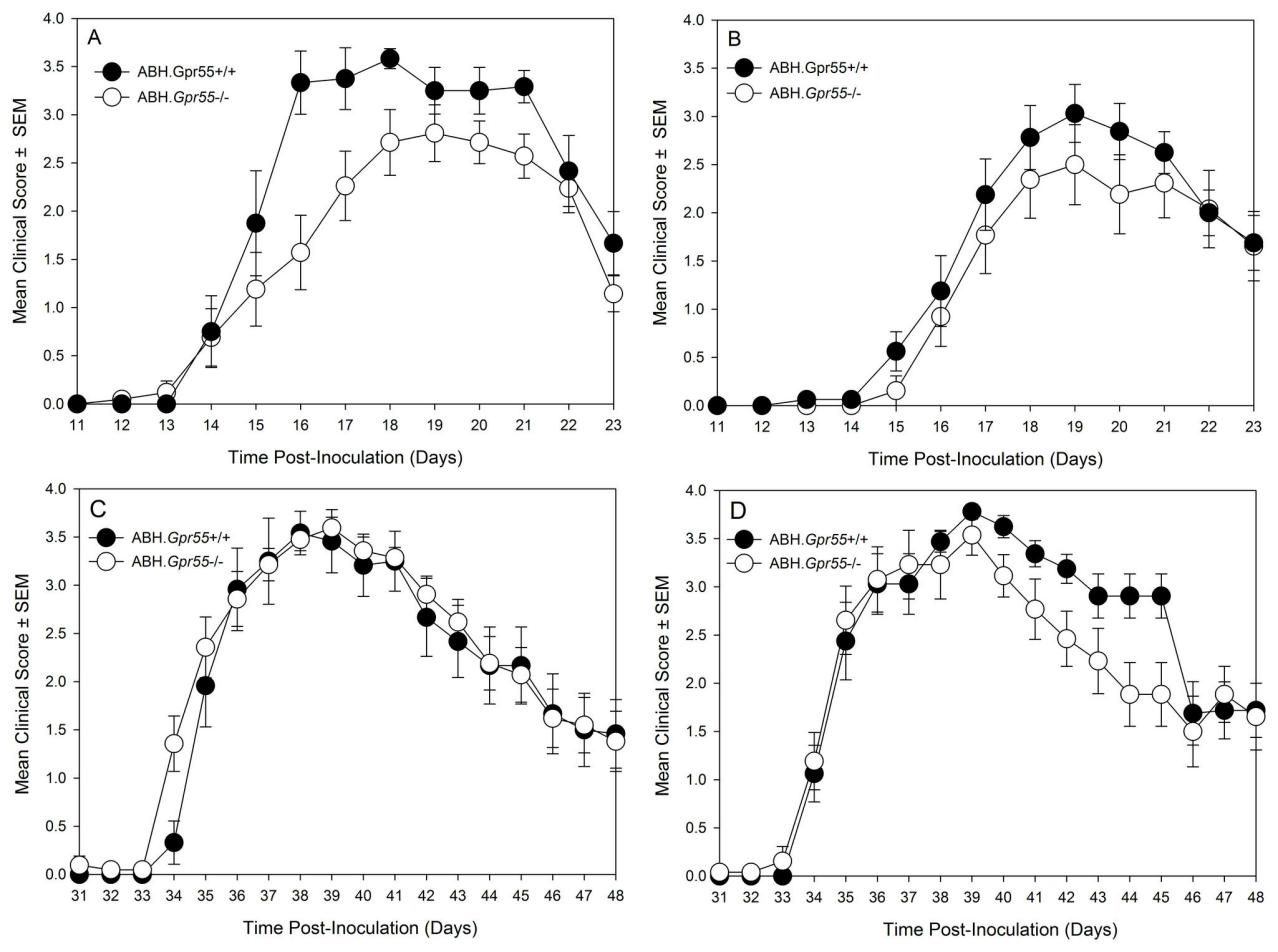
GPR55-deficiency has marginal effects on the development of EAE in ABH mice. (A, C) Female and (B, D) male ABH wildtype (black circles) or ABH.*Gpr55*-knockout mice (white circles) were injected with spinal cord homogenate in Freunds adjuvant on day 0 and 7 and a relapse was induced on day 28 post-inoculation. Neurological signs were scored daily 0-5 scale. The results show the disease course during (A, B) the initial acute or (C, D) an induced relapse. The results represent the mean ± SEM daily scores. n = 12-21/group.

These studies indicate that genetic background can influence the disease course. Therefore, we backcrossed the GPR55 gene deletion for over 11 generations onto the ABH genetic background to generate fully congenic ABH.*Gpr55*-/- mice. Following the induction of EAE in these mice there was a marginal inhibitory effect on the clinical course in the initial acute phase of EAE (Figure 4A), with a small but statistically significant (P<0.05) reduction in the severity of EAE in female mice (Table 1). There was no influence on disease in male mice (Figure 4B and Table 1G). However, when a relapse was induced in these mice there was no inhibitory effect in either female or males mice (Figure 4C & 4D and Table 1H). This suggests that antagonism of the GPR55 may offer little as a means of immunosuppressing disease and was consistent with the lack of any obvious phenotypic differences following T and B cell immunophenotying in GPR55-deficient, C57BL/6 mice. This suggests that genetic background and disease induction can have major influence on outcome in the prediction of the influence of transgenesis during EAE.

## Discussion

There has been a general failure to translate findings from animal models into the treatment of many human diseases. This includes treatments of stroke and multiple sclerosis [31,32]. Part of the problem is probably due to failings with the clinical development of agents such as inappropriate trial design and patient selection. Failure to translate treatments may relate also to differences in the biology between rodents and humans or the lack of validity of the animal models. Importantly, studies in rodents seldom examine pharmacological agents in a therapeutic context and at a pharmacological dose relevant to how they will be used clinically [32]. As such, a therapeutic treatment paradigm in rodents may consist of treatment for a few days after the development of first signs compared to the months and years in humans following development of relapsing disease [32]. It is has also been noted that reporting and possibly implementation of elements of experimental design have been poor in animal studies and that may contribute to the lack of translation [31-33]. An additional potential problem as highlighted here could be the use of inbred animals in research. This means that drugs are being tested in essentially hundreds of the same individual. This may be a particular problem if the standard tool has poor consistency and translatability. Although one solution may be to use outbred mice, this adds to variability and increases group sizes, which is against the principles of reduction, refinement and replacement of animals in research. Therefore, it has been argued that replication in a number of inbred strains may be useful [34]. Whilst there may be debate about which animal strains better reflect human disease, reproduction of findings across a number of strains and species may increase the translational value. As such, FTY720 consistently inhibits development of EAE across a number of strains and species [35,36] and formulated FTY720 in the form of fingolimod inhibits relapsing MS in humans [37].

Myelin peptide-induced disease is useful for the study of T cell immunology *in vitro*, but this may not always give the reproducibility of disease induction as found using tissue homogenates [16,30]. The disease course in spinal cord homogenate-induced disease in ABH mice has been consistent over many years [16,21]. Such reproducibility means that failure to show high incidence of robust severity and compact timing of onset is a failure in the quality-control of the experiment, which would need repeating. In contrast there appears to be marked variability in the degree of susceptibility to MOG-induced disease in C57BL/6 mice. This has occurred in these experiments, but different disease courses can occur between and even within the same publications. This variability seen in disease course could sometimes mean that the influence of the transgene has as much to do with the disease incidence and severity of the control group. It is not uncommon to find weak disease in controls when the transgene or treatment appears to exacerbate disease, yet control animals develop strong disease when the transgene or treatment appears to inhibit disease. This lack of consistency of disease often gets missed during the review process, but may help contribute to the dogma about the importance of the therapeutic target. Line graphs are often used in reporting of EAE to show the development of disease over time. Low scores in graphs can mean that animals had lower severity disease in general or it could mean a low incidence of severe EAE. Thus, without reporting of incidence of disease, group sizes and severity and variability the disease induced, as occurs commonly, this means it is often impossible to interpret the data [30,32]. This was shown here (Figure 1, Figure 2A) by way of example and reporting and refereeing standards need to be improved [32,38].

The data from C57BL/6 background CB_2_ receptor and GPR55 knockout mice were used in this study to provide a concrete example of how the control group could influence the result, especially as some of the studies in GPR55 knockout mice were performed at the same time and with the same batch of immunizing adjuvant as the study in the CB_2_ knockout mice. However, vastly different responses were seen in control groups. The influence of loss of GPR55 protein was supported in additional experiments in C57BL/6 background mice. The finding that CB_2_ receptor knockout can lead to enhanced disease severity in C57BL-background Cnr2-/- mice [12,13] was thus consistent in C57BL/6.*Cnrt* ^*tm1Zim*^ and C57BL/6.*Cnr2* ^*tm1Dgen*^ mice. However, this enhancing effect was not noticeable in ABH.*Cnr2*-/- knockout mice, once the CB_2_ receptor knockout was generated on a fully EAE-susceptible genetic background. This may relate to the high severity of disease that is induced in ABH mice, but the data supports the observation that CB_2_ receptor agonists or antagonists had no influence on the development of autoimmunity in ABH mice [16]. This is perhaps not surprising as it was found that marked immunosuppression only occurs when cannabimimetic effects are induced following stimulation of neural CB_1_ receptors, not immune cells [12,18]. Whilst weak disease may allow a small inhibitory effect or augmentation to be seen, when severe disease is induced, it may mean that any small inhibitory/stimulatory effect is masked, as occurred here. However, with such minor differences resulting in a small delay or small reduction in severity of disease, then the chances of the results being medically relevant are markedly decreased. Importantly, the chances of pharmacological inhibition of disease, translating into human benefit are probably reduced also. 

Whilst it has been reported that disease severity was enhanced in B6.*Trpv1-/*- and B6.*Cnr1*-/- mice [10,14], these effects were not particularly noticeable in congenic ABH.*Cnr1* and ABH.*Trpv1* knockout mice seen here or reported previously [9]. CB_1_ receptor mice poorly tolerate the immune insult and accumulate nerve damage and residual deficit following neurological attack. This is consistent with a neuroprotective role of these molecules and is seen with poor recovery from attack during EAE [32], rather than an overt immune-enhancing effect [9,18,32]. Enhanced severity and poor recovery from EAE has been reported in C57BL/6.*Trpv1*-/- [14], but recently it has been reported that B6.*Trpv1*-/- do not develop MOG_35-55_ peptide induced EAE suggesting some immune influence [39], despite animals being obtained from the same source and using essentially the same EAE induction technique. This further highlights the inconsistency of MOG peptide-induced EAE that can sometimes occur in C57BL/6 mice [14,39]. Furthermore, immunomodulatory influences in B6.*Faah*-/- fatty acid amide hydrolase (FAAH) knockout mice have been suggested by the disease course [10], but have not been supported by other studies in either B6.*Faah* ^*-/-*^ [40,41] or ABH.*Faah*-/- [41] mice. In both instances, the data suggest that FAAH may limit neurodegeneration as a consequence of immune attack, which is consistent with pharmacological control of disease by exogenous cannabinoids [9,18]. During EAE in ABH mice the initial attack is driven by an immune-mediated effect, whereas the relapse additionally contains demyelination and neurodegenerative effects and it is more easy to dissociate immunosuppressive verse non-immunosuppression related neuroprotective effects [16,21,42]. This is more difficult in C57BL/6 mice as EAE is typically monophasic and is neurodegenerative from onset [10,14,42]. Based on the common findings in these cannabinoid knockout mice, it is suggests that some of the immune phenotypes seen in many C57BL/6 knockout studies may likewise become marginal if the studies were performed in other genetic backgrounds

In comparison to SJL/J and ABH mice, C57BL/6 mice are relatively EAE resistant to disease induction with spinal cord autoantigens and this genetic resistance to disease may account for variable disease onset [15,16]. Furthermore, this inconsistency in disease induction may relate also to the use of MOG_35-55_ peptide for immunization. MOG_35-55_ was first found to induce EAE in Biozzi ABH mice, where it also induces chronic EAE [30,42]. However the MOG_35-55_ epitope is a subdominant compared to MOG_8-22_ in ABH mice and induces inconsistent disease incidence, which is sometimes subclinical [30]. In one experiment, MOG_35-55_ peptide induced an EAE score of 3.3 ± 0.8 and day of onset 22.8 ± 5.7 (range 16-30 n=5) in ABH mice, compared to much narrower range of day of onset in spinal cord induced disease, as shown here. A large range in timing of disease onset tends to skew the data to incorrectly appear progressive in nature [32]. Likewise, there are a number of immunogenic and pathogenic epitopes in MOG for C57BL/6 mice [43,44]. It has suggested that MOG_35-55_ may also be a subdominant encephalitogen in C57BL/6 mice compared to MOG_119-132_ [45]. This too may influence the degree of EAE-susceptibility in C57BL/6 mice as is can in ABH mice [30,42]. To avoid this potential inconsistency we have backcrossed our C57BL/6 transgenic mice onto the ABH mouse background, however consistency also may be enhanced through the use of commercial, quality-controlled adjuvant and adopting standards for the severity and onset of disease in control groups. Alternatively, the lack of influence of cannabinoid gene knockouts in Biozzi ABH mice compared to that found or reported in EAE in C57BL/6 may relate to the paucity of polymorphonuclear neutrophils in ABH mouse EAE [16,46] as occurs in MS [47], compared to more marked neutrophil involvement in EAE in C57BL/6 mice [48], as occurs in Devics MS/neuromyelitis optica [47]. Subtle variations in cellular and humoral immunopathological effector mechanisms between C57BL/6 and Biozzi ABH mice and the differences induced by MOG_35-55_ and spinal cord homogenate probably contribute to the differences between the two different EAE models [49-51].

This study demonstrated marked differences in the susceptibility to EAE in different C57BL/6 lines of mice in one experiment two different control lines showed marked differences in susceptibility (Table 1A). This may in part relate to the influences of 129 and C57BL/6 genes to EAE susceptibility. At the time of testing the wildtype GPR55 knockout line would have had more background 129 genes than the low-susceptibility CB2 knockout line suggesting that they may harbour more susceptibility loci or fewer resistant loci, as 129 mice may be slightly more susceptible than C57BL/6 to MOG35-55-induced EAE. These genes and copy number can affect the level of immune response especially if the gene deletion is located near a susceptibility locus [52,53]. That the lines gave consistent disease suggests that differences may be genetic rather than random variation in susceptibility. It is known there are subtle genetic differences between C57BL/6 sublines [54] and different sublines of mice can sometimes show differences in susceptibility to EAE [55]. Therefore we used appropriate littermate controls for these experiments. However, when backcrossed onto the ABH mouse background and spinal cord homogenate was used to induce disease there was much more consistent disease. The low level of susceptibility in some C57BL/6 mouse experiments suggests that it is important to set quality control elements in disease susceptibility when performing such experiments

In the cannabinoid field, few agents are specific for their target and cannabinoid ligands have variable degrees of selectively for their receptors [11]. Furthermore agonists and antagonists can have off-target effects such as SR141617A which has both an influence on CB_1_ receptor and GPR55, whereas other agents such as O-1602 may bind to GPR55 and GPR18 [3,56]. Therefore, use of gene knockout mice is an excellent complementary tool to validate potential targets of therapeutic agents. However, they may not be infallible. Although in many instances the entire coding gene can be removed, in other transgenic mice, sections of the gene are replaced with a neomycin resistance targeting cassette to functionally inactivate the gene. In the Cnr2^tm1Dgen^ mouse there is a 391 base pair deletion in the N terminus of *Cnr2*. In contrast, the Cnr2^tm1Zim^ mice have the neomycin resistance cassette inserted into the last 341 nucleotide base pairs of the C terminal end of the coding *Cnr2* exon. They therefore only lack a portion of the intracellular loop 3, transmembrane domains 6 and 7, and the carboxy terminus, which is sufficient to functionally inactivate the CB_2_ receptor [27]. However, these mice have an intact N terminus and promoter of the *Cnr2* gene and produce a truncated CB_2_ receptor message (Nephi Stella. University of Washington, Seattle, USA, Personal Communication). This may produce some dysfunctional protein, as a cytomegalovirus promoter driven construct of the predicted truncated CB_2_ gene in Cnr2^tm1Zim^ mice from the N terminus to the stop codon after the targeting cassette, led to detectable protein expression in the cytosol, but not the surface, of transfected HEK293 cells (Ken Mackie and Brady Atwood. University of Indiana, Bloomington, USA. Personal communication). That the relaxation effect of (R)3-(5-dimethylcarbamoyl-pent-1-enyl)-N-(2-hydroxy-1-methyl-ethyl)benzamide, which does not bind to CB_2_ receptor was inhibited in Cnr2^tm1Zim^ but not in Cnr2^tm1Dgen^ mice suggests that some inhibitory molecule may be indeed generated in this mouse and possibly interfere with GPR55 function. Whilst the molecular nature of the precise difference between the strains is unknown, the pharmacologically different response is highlighted here, to alert people using these mice. It is interesting that it has been found that GPR55 can influence CB_2_-mediated effects, via “cross-talk” effects on cell signalling [57]. Therefore, Cnr2^tm1Zim^ mice may functionally influence other signalling pathways in addition to those of CB_2_ receptors. Therefore investigators should be cautious when interpreting phenotypes using this mouse line, especially as CB_2_ and GPR55 may influence similar functions such as bone formation and neutrophil function [58-60]. For this reason Cnr2^tm1Dgen^ mice were used to generate congenic *Cnr2*-deficient mice.

It was evident that there were strain differences in susceptibility to EAE induction and whilst it is believed that the differences were related to the genetic deletion, it is recognised that as the cells used to generate these knockout mice were chimeras of 129 and C57BL/6 mice, it is therefore likely that genetic elements of 129 or C57BL/6 mice are in linkage disequilibrium with the gene silencing cassette, despite extensive backcrossing. This associated genetics can sometimes influence or even account for the immune phenotype [53,54]. Susceptibility to EAE is polygenic [61-66] and susceptibility loci have been mapped nearCnr1 (chromosome 4. 16cM), Cnr2 (chromosome 4. 68cM), Trpv1 (chromosome 11. 45cM) and Gpr55 (chromosome1 44 cM). Minor, non-major histocompatibility complex loci influencing susceptibility and severity of disease have been mapped to regions including chromosomes 4 and 11 in ABH mice, although the major genes controlling susceptibility map to chromosome 7 [62,64]. Both 129 and C57BL/6 are relatively EAE resistant [67] but the C57BL/10 genetic background, which is related to C57BL/6 but contains differences on chromosome 4 amongst others [68], has been used in mapping studies and has been shown to contain EAE susceptibility and resistance genes. Major loci are mapped to chromosome 3 and 7 but many others have been identified across the genome of C57BL background mice [61,63,65,66]. These include loci on chromosomes 4 and 11 [65] and interestingly an EAE susceptibility loci in female mice maps to chromosome 1 (70-90cM) in C57BL (C57BL/10.RIII) background mice [66]. Whether this influenced susceptibility in GPR55 knockout mice is unknown, but must be borne in mind when considering the data. However, the differences in susceptibility reported between C57BL/6.Trpv1 are unlikely to relate to genetics as the animals were from the same source, indicating that variation in disease induction can occur [14,39]. At the time of study there were no specific, high affinity GPR55 antagonists available to confirm the influence on disease course. However that CB_2_ receptor agonism and antagonism did not influence EAE susceptibility in ABH mice [18], whereas CB_2_ antagonism augmented disease in C57BL/6 mice [69], the pharmacological approaches of receptor antagonism are consistent with the influence of genetic depletion of receptors in both strains suggesting that the effect is due to the gene targeting and not adjacent chromosomal regions. 

Deletion of cannabinoid receptor genes have not been associated with a sexually dimorphic effects in EAE, yet it was apparent that GPR55 deficient female mice may develop less autoimmunity compared to males and was seen in both C57BL/6.*Gpr55*-/- and ABH.*Gpr55*-/- mice. The molecular mechanism of this was not found, but no obvious immune T cell phenotype was detected here. GPR55 may be involved in macrophage function and antigen-presentation as GPR55 is reported to influence osteoclast function [59]. However, the immune influence may be downstream of an influence of sex hormones between GPR55 knockout and wildtype mice. Gonadal hormones are known to influence susceptibility to EAE [70] and previously gender-restricted and sex hormonal effects have been noted in GPR55-knockout mice [4,60,71]. Although more females develop MS than males [72], studies in EAE show that this can be a complex relationship with females sometimes being more susceptible to males and *vice versa* [70,73-75]. Likewise other gender and strain hormonal influences such as differences in calcifediol a vitamin D prehormone occur between ABH and C57BL/6 mice, which again could influence immunity as vitamin D response elements may control a number of susceptibility genes including MHC expression [76,77]. However, the GPR55-mediated effect in female mice was limited in ABH mice. At the time of these studies there were no high affinity, specific GPR55 antagonists available to investigate this further. It has been suggested that cannabidiol may act as a GPR55 antagonist [78], amongst other things, and whilst it has been reported that cannabidiol can affect EAE in C57BL/6 mice [79], it has no influence on the autoimmune component of EAE in ABH mice [12]. In contrast high doses (>2.5mg/kg i.p.) of THC could cause immunosuppression in wildtype and CB_2_-deficient mice but not to any appreciable extent in CB_1_-deficent mice. This further supports the CB_1_-receptor mediated immunosuppressive effect that we and others identified earlier previously in ABH and C57BL/6 mice [18,80]. However, we believe that a T cell immunosuppressive effect of cannabinoids in mice, notably THC, is probably an artefact of suprapharmacological/supraphysiological doses of THC that cause sedative side-effects in animals, which will never be achieved in humans. Doses that do not cause overt cannabimimetic signs are not immunosuppressive and do not inhibit the generation of EAE, in mice, yet can be useful for symptom control of spasticity that occurs as a consequence of damage from repeated neurological attacks [7,18]. This is consistent with the observations that THC had no real influence on immune function during phase III trials of cannabis and THC for symptom control [81]. However, whilst cannabinoids in our opinion may be not be that important for the generation of relapsing autoimmunity, once generated cannabinoids may have neuroprotective effects via an action on microglial cells and nerves to limit the consequences of immune attack, facilitating better recovery [1,9,10,18,82]. This is in addition to their proven effects on symptom control both in EAE and MS [7,8,83]. In conclusion this study shows that the influence of transgenesis can vary, dependent on a number of factors. However, one factor that needs to be addressed is ensuring that there is robust and consistent disease in transgenic/gene knockout studies to help improve the translational hit rate of animal studies. 

## Supporting Information

Figure S1 **GPR55-deficiency has no effect on mitogen or MOG-induced proliferation in C57BL/6 mice.** Female C56BL/6.*Gpr55* knockout (KO) and heterozygous littermates expressing the wildtype (WT) GPR55 gene were immunized with MOG_35-55_ peptide in Freunds adjuvant on day 0 and were injected with 200ng of *B. pertussis* toxin on day 0 and 1. L were collected on day 9 and re-stimulated *in* *vitro* with either (A) 1μg concanavalin A for 48h (B) MOG peptide at concentrations 1μg or 10μg for 72h (A). A total of 300,000 cells were resuspended in a final volume of 100 μl of RPMI medium containing 10% foetal calf serum and plated in 96 well-plates. After 24-48h a total of 0.5 units of ^3^H Thymidine (PerkinElmer LAS, Beaconsfield, Bucks, UK) was added to each well and cells were incubated during for 24h at 37°C in 5%CO_2_. Cells were then harvested (TOMTEC MACH III M CELL HARVESTER 96, Warwick, UK) and analysed on a counter (Wallac 1450, Microbeta Plus Liquid Scintillation Counter, Cambridgeshire, UK).
(PDF)

Figure S2 **GPR55-deficiency has no effect on MOG proliferation in vivo in C57BL/6 mice.** C56BL/6.*Gpr55* knockout and wildtype female littermates were immunized with MOG_35-55_ peptide in Freunds adjuvant on day 0 and were injected with 200ng of *B. pertussis* toxin on day 0 and 1. Lymphocytes were collected on day 9 and left either unstimulated or were re-stimulated *in* *vitro* with MOG peptide at a concentrations of 10μg/mg for 72h. n = 3/group. Cells were incubated with CSFC and the resultant cellular proliferation assessed using the number of generations by flow cytometry. Results present the mean + SEM. n=3/group.
(PDF)

Figure S3 **CB_2_ receptor knockout variants demonstrate different pharmacological responses to a GPR55 modulator.** The vasa deferentia from male C57BL/6 mice and (A) C57BL/6.*Gpr55* ^*tm1Tigm*^ or (B) C57BL/6.*Cnr1* ^*tm1Zim*^, C57BL/6.*Cnr2* ^*tm1Zim*^, C57BL/6.*Cnr2* ^*tm1Dgen*^ were electrically stimulated the contraction responses assessed following addition of various concentrations of (R)3-(5-dimethylcarbamoyl-pent-1-enyl)-N-(2-hydroxy-1-methyl-ethyl) benzamide the inhibition assessed. The results represent the mean ± SEM contractions n=5-6/group.
(PDF)
