# Supplementary figures and images for: Genetic Background Can Result in a Marked or Minimal Effect of Gene Knockout (GPR55 and CB2 Receptor) in Experimental Autoimmune Encephalomyelitis Models of Multiple Sclerosis
Source: PLoS One. 2013 Oct 9;8(10):e76907. doi: 10.1371/journal.pone.0076907 (PMC3793915; doi:10.1371/journal.pone.0076907)

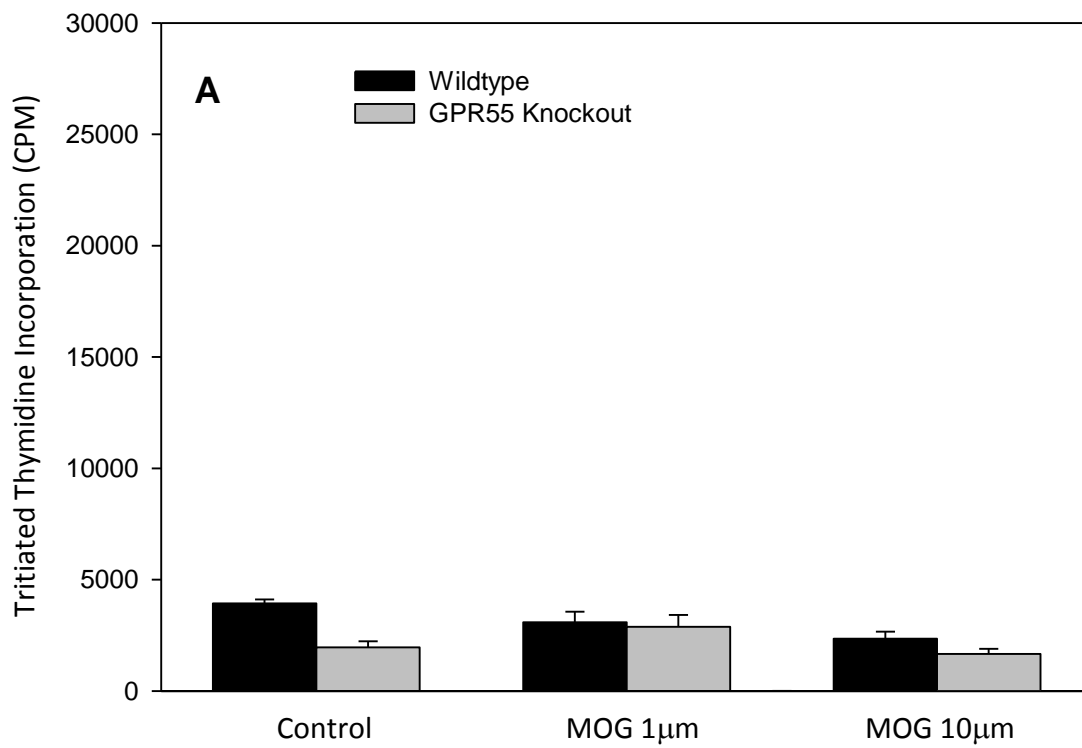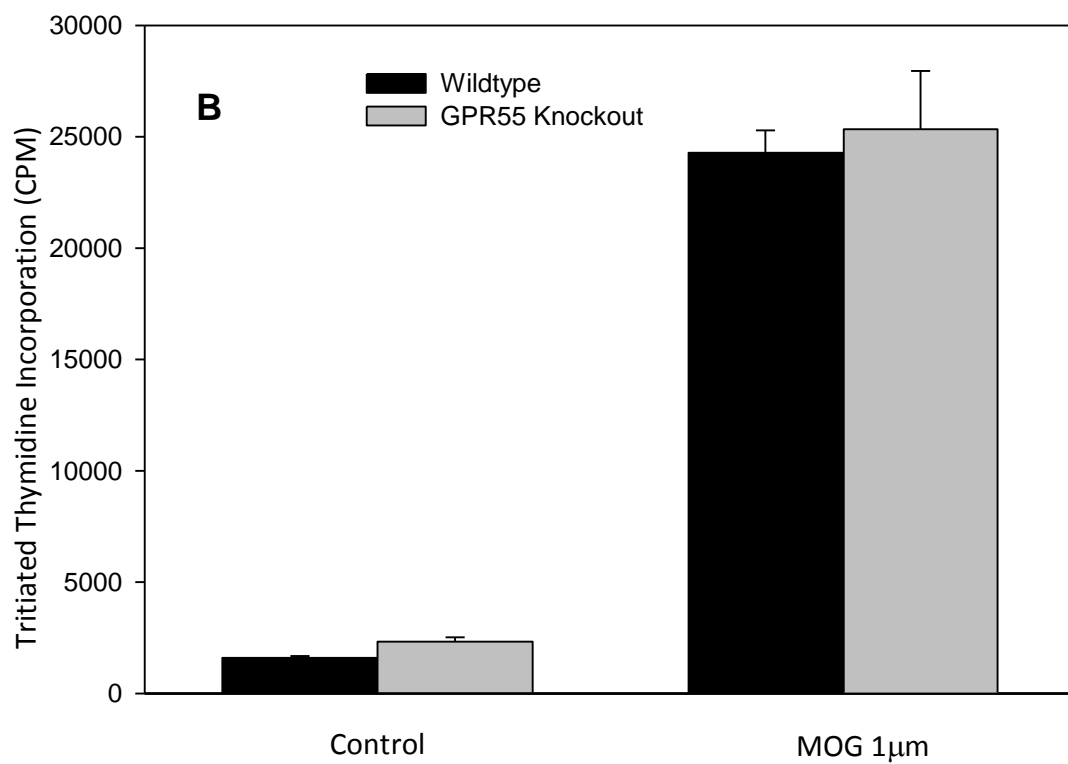

Supplement: Figure S1 — GPR55-deficiency has no effect on mitogen or MOG-induced proliferation in C57BL/6 mice. Female C56BL/6.Gpr55 knockout (KO) and heterozygous littermates expressing the wildtype (WT) GPR55 gene were immunized with MOG35-55 peptide in Freunds adjuvant on day 0 and were injected with 200ng of B. pertussis toxin on day 0 and 1. L were collected on day 9 and re-stimulated in vitro with either (A) 1μg concanavalin A for 48h (B) MOG peptide at concentrations 1μg or 10μg for 72h (A). A total of 300,000 cells were resuspended in a final volume of 100 μl of RPMI medium containing 10% foetal calf serum and plated in 96 well-plates. After 24-48h a total of 0.5 units of 3H Thymidine (PerkinElmer LAS, Beaconsfield, Bucks, UK) was added to each well and cells were incubated during for 24h at 37°C in 5%CO2. Cells were then harvested (TOMTEC MACH III M CELL HARVESTER 96, Warwick, UK) and analysed on a counter (Wallac 1450, Microbeta Plus Liquid Scintillation Counter, Cambridgeshire, UK). (PDF) [file pone.0076907.s001.pdf]

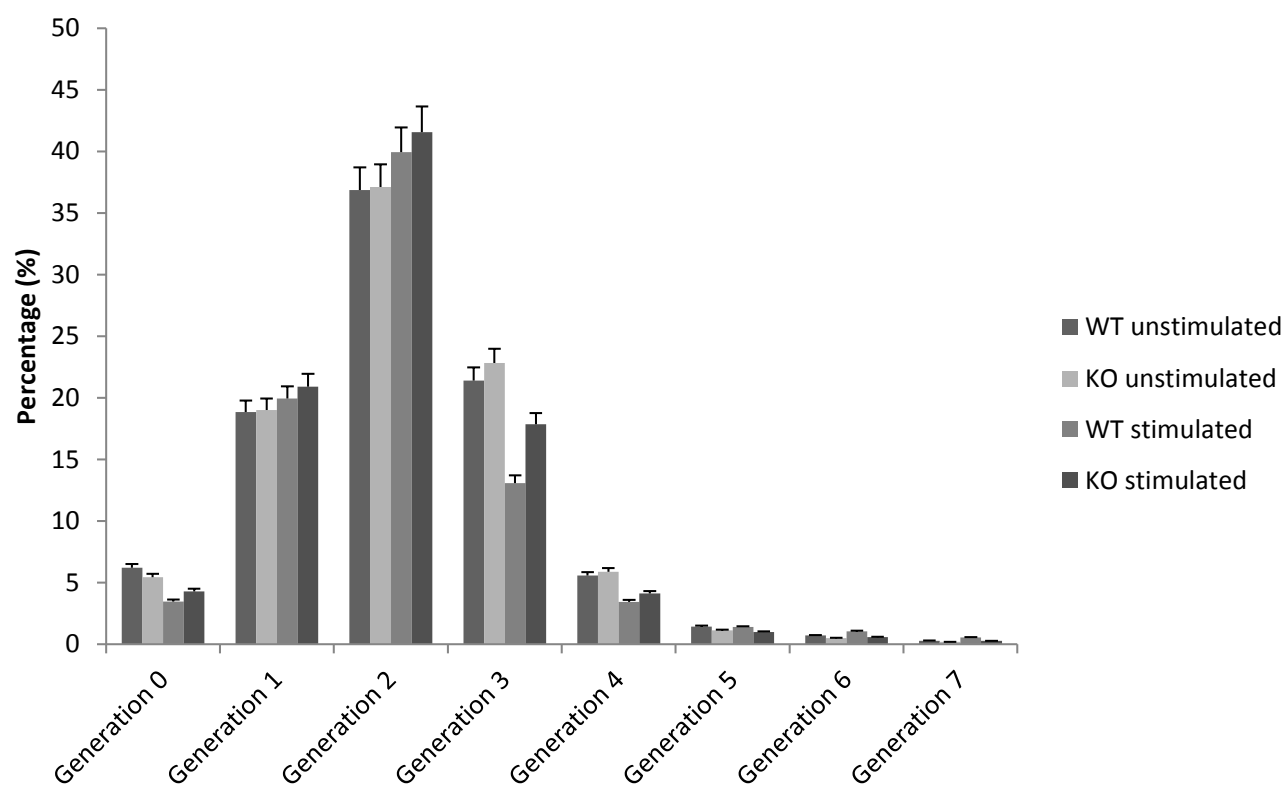

Supplement: Figure S2 — GPR55-deficiency has no effect on MOG proliferation in vivo in C57BL/6 mice. C56BL/6.Gpr55 knockout and wildtype female littermates were immunized with MOG35-55 peptide in Freunds adjuvant on day 0 and were injected with 200ng of B. pertussis toxin on day 0 and 1. Lymphocytes were collected on day 9 and left either unstimulated or were re-stimulated in vitro with MOG peptide at a concentrations of 10μg/mg for 72h. n = 3/group. Cells were incubated with CSFC and the resultant cellular proliferation assessed using the number of generations by flow cytometry. Results present the mean + SEM. n=3/group. (PDF) [file pone.0076907.s002.pdf]

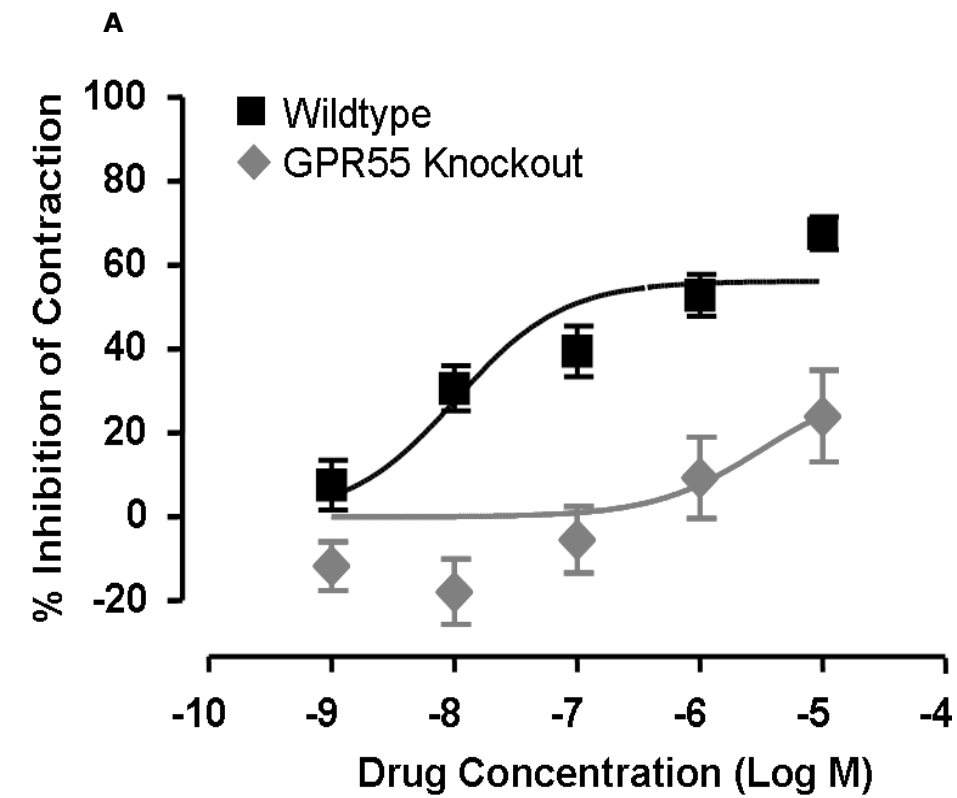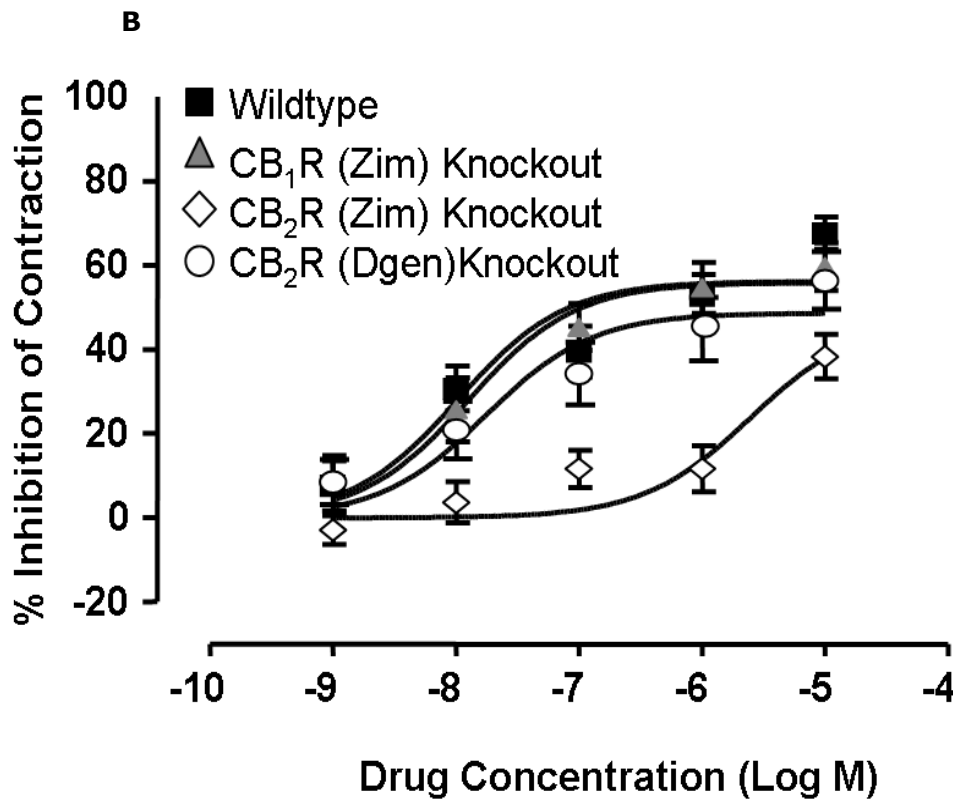

Supplement: Figure S3 — CB2 receptor knockout variants demonstrate different pharmacological responses to a GPR55 modulator. The vasa deferentia from male C57BL/6 mice and (A) C57BL/6.Gpr55 tm1Tigm or (B) C57BL/6.Cnr1 tm1Zim, C57BL/6.Cnr2 tm1Zim, C57BL/6.Cnr2 tm1Dgen were electrically stimulated the contraction responses assessed following addition of various concentrations of (R)3-(5-dimethylcarbamoyl-pent-1-enyl)-N-(2-hydroxy-1-methyl-ethyl) benzamide the inhibition assessed. The results represent the mean ± SEM contractions n=5-6/group. (PDF) [file pone.0076907.s003.pdf]
